# Supplementary material for: Interrogating Emergent Transport Properties for Molecular Motor Ensembles: A Semi-analytical Approach
Source: PLoS Comput Biol. 2016 Nov 3;12(11):e1005152. doi: 10.1371/journal.pcbi.1005152 (PMC5094777; doi:10.1371/journal.pcbi.1005152)
Supplement: S1 Text — (PDF) [file pcbi.1005152.s001.pdf]

Supplementary Information Text S1

Maximum distance between the vanguard and rearguard motor

Here, the validity of Result 1 is established.
Result 1 : Given an ensemble of M molecular motors attached to a common cargo that is subjected to a load force F_{load}, the distance between the rearguard and the vanguard motor is bound by

$$n = max\left\{\frac{(M+1)max(F_s,\bar{F}_s) - F_{load}}{K_e} + d, \frac{F_{load}}{K_e}\right\} + 2L_0$$

where F_s is the minimum load force for which the stepping probability of the wild-type motor protein becomes zero (i.e. the stalling force for the wild-type motor protein), $\bar{F}_s$ is the minimum load force for which the stepping probability of the mutant motor protein becomes zero (i.e. the stalling force for the mutated motor protein), L_0 is the rest length of the motor linkage and K_e is the linkage stiffness.

Consider an ensemble of wild-type and mutant motors bound to a cargo with the following assumptions :

- There are a total of M (wild-type and mutant) motors bound to the cargo. The wild-type motor stalling force is F_s and the mutant motor stalling force is $\bar{F}_s$.
- The motor linkages have a rest length of L_0 and stiffness K_e. The linkages are modelled as hookean springs when stretched that offer no resistance when compressed.
- There exists a constant load force F_{load} on the cargo.
- Z_{eq} is the mean position of the cargo, which is the equilibrium position determined by the forces exerted on the cargo by the motors through their linkages and the load force F_{load}
- Any unattached motor bound to the cargo can attach to only those locations on the microtubule that are within L_0 distance of the cargo position.

For the discussion below, it is assumed that there is at least one motor on the cargo opposing its motion (for other cases, similar arguments can be used). Suppose there be f motors assisting the cargo motion and r motors opposing it ($f + r \leq M$). Let the locations of the f motors on the microtubule assisting the cargo motion be $Z_1^+, Z_1^+, ..., Z_f^+$. The linkage length of a motor located at Z_i^+ is given by $L_i^+ = Z_i^+ - Z_{eq}$ and the force it exerts on the cargo is $F_i^+ = K_e(L_i^+ - L_0)$. If the motors are located in such a way that $0 < L_0 \leq L_1^+ \leq L_2^+ \leq ... \leq L_f^+$, then $F_1^+ \leq F_2^+ \leq ... \leq F_f^+$ and the total force exerted on the cargo in the forward direction is $F_{fwd} = \sum_{i=1}^{f} F_i^+$.

Let the locations of the r motors on the microtubule opposing the cargo motion be $Z_1^-, Z_1^-, ..., Z_r^-$. The linkage length of a motor located at Z_j^- is given by $L_j^- = Z_{eq} - Z_j^-$ and the force it exerts on the cargo is $F_j^- = K_e(L_j^- - L_0)$. If the motors are located in such a way that $0 < L_0 \leq L_1^- \leq L_2^- \leq ... \leq L_r^-$, then $F_1^- \leq F_2^- \leq ... \leq F_r^-$ and the total force exerted on the cargo in the backward direction is $F_{back} = \sum_{j=1}^{r} F_j^-$. The separation between the vanguard and rearguard motor can be found as $S = L_f^+ + L_r^-$ where L_f^+ and L_r^- are the linkage lengths of the vanguard and rearguard motor respectively.

Suppose the vanguard motor (thereby all the motors) is not stalled. Then $F_{fwd} < M\ max(F_s, \bar{F}_s)$. If the vanguard motor is a wild-type motor, $F(L_f^+) < F_s$ thus $L_f^+ < \frac{F_s}{K_e} + L_0$ and if it is a mutant motor, $F(L_f^+) < \bar{F}_s$ thus $L_f^+ < \frac{\bar{F}_s}{K_e} + L_0$. In general $L_f^+ < \frac{max(F_s,\bar{F}_s)}{K_e} + L_0$. At equilibrium, $F_{back} = F_{fwd} - F_{load}$, thus $\sum_{j=1}^{r} F_j^- = F_{fwd} - F_{load}$ which implies that $F(L_r^-) + \sum_{j=1}^{r-1} F_j^- = F_{fwd} - F_{load}$.

It follows that $F(L_r^-) \leq F_{fwd} - F_{load}$. Substituting for $F(L_r^-)$ it follows that $K_e(L_r^- - L_0) < M\ max(F_s, \bar{F}_s) - F_{load}$ and thus $L_r^- < \frac{M\ max(F_s,\bar{F}_s) - F_{load}}{K_e} + L_0$. Thus the separation S between the vanguard and rearguard motor when none of the motors are stalled is given as,

$$S < \frac{(M+1)max(F_s,\bar{F}_s) - F_{load}}{K_e} + 2L_0. \tag{1}$$

Let $n_{max} := \frac{(M+1)max(F_s,\bar{F}_s)-F_{load}}{K_e} + d + 2L_0$. Thus, if none of the motors are stalled then the separation $S < (n_{max} - d)$.

Consider a current configuration where the separation $S' < n_{max}$. In the current configuration, the vanguard motor is either in stalled (Case I) or not stalled (Case II) condition.

Case I : If the vanguard motor is stalled, then any ensuing change in the configuration will only decrease the separation since the vanguard motor cannot step forward and the rearguard motor cannot step backward. Thus the separation remains less than n_{max}.

Case II : If the vanguard motor is not stalled, then using (1) it can be stated that the separation $S' < (n_{max} - d)$. Any change in the current configuration that can increase the separation is if the vanguard motor takes a forward step of length d, where the new separation $(S' + d) < n_{max}$. Thus the separation remains less than n_{max}.

It can thus be stated that if in a current configuration the separation is less than a bound n_{max} then for any subsequent configuration the bound is obeyed.
