# Supplementary material for: Interrogating Emergent Transport Properties for Molecular Motor Ensembles: A Semi-analytical Approach
Source: PLoS Comput Biol. 2016 Nov 3;12(11):e1005152. doi: 10.1371/journal.pcbi.1005152 (PMC5094777; doi:10.1371/journal.pcbi.1005152)
Supplement: S2 Text — (PDF) [file pcbi.1005152.s002.pdf]

Supporting Information Text S2

Transition rates between relative configurations

Here the validity of Result 2 is established.
Result 2 : The rate of transition between relative configurations ϑ *and* ϑ' *is given by*

$$\nu_{\vartheta}(\vartheta', \vartheta) = \sum_{0 \leq \beta \leq \lceil \frac{n}{d} \rceil} \nu_{\Omega}(\tau^{\beta}\Omega', \Omega),$$

where $\Upsilon(\Omega) = \vartheta$, $\Upsilon(\Omega') = \vartheta'$, $(\tau^{\beta}\Omega')$ *is an absolute configuration obtained after linearly shifting all the motors in* Ω *by* β *locations on the microtubule towards the right and* d *is the periodicity of the microtubule lattice*

Let Ω and Ω' be a pair of absolute configurations such that $\Upsilon(\Omega) = \vartheta$ and $\Upsilon(\Omega') = \vartheta'$. It is assumed that the system is translation invariant with its stochastic behavior unaffected if the cargo and the ensemble of motors bound to it shift to a new location along the microtubule. The translation invariance property enables the construction of a set $\Omega(\vartheta)$ of all absolute configurations that have the same relative configuration ϑ as $\Omega(\vartheta) = \{\tau^{\beta}\Omega : \beta \in I\}$, where I is the set of integers. In a similar manner, $\Omega(\vartheta') = \{\tau^{\beta'}\Omega' : \beta' \in I\}$

Using the definition of transition probability, $P_{\vartheta}(\vartheta', t + \Delta t|\vartheta, t)$ can be expressed as follows :

$$\begin{aligned}
P_{\vartheta}(\vartheta', t + \Delta t|\vartheta, t) &= \frac{P_{\vartheta}(\vartheta', t + \Delta t, \vartheta, t)}{P_{\vartheta}(\vartheta, t)} \\
&= \frac{\displaystyle\sum_{\Omega \in \Omega(\vartheta)} \sum_{\Omega' \in \Omega(\vartheta')} P_{\Omega}(\Omega', t + \Delta t, \Omega, t)}{\sum_{\Omega \in \Omega(\vartheta)} P_{\Omega}(\Omega, t)} \\
&= \frac{1}{\sum_{\beta} P_{\Omega}(\tau^{\beta}\Omega, t)} \quad \sum_{\beta} \sum_{\beta'} P_{\Omega}(\tau^{\beta'}\Omega', t + \Delta t, \tau^{\beta}\Omega, t) \\
&= \frac{1}{\sum_{\beta} P_{\Omega}(\tau^{\beta}\Omega, t)} \quad \sum_{\beta} \sum_{\beta'} P_{\Omega}(\tau^{\beta'}\Omega', t + \Delta t|\tau^{\beta}\Omega, t) P_{\Omega}(\tau^{\beta}\Omega, t) \\
&= \frac{1}{\sum_{\beta} P_{\Omega}(\tau^{\beta}\Omega, t)} \sum_{\beta} P_{\Omega}(\tau^{\beta}\Omega, t) \ \sum_{\beta'} P_{\Omega}(\tau^{\beta'}\Omega', t + \Delta t|\tau^{\beta}\Omega, t) \\
&= \frac{1}{\sum_{\beta} P_{\Omega}(\tau^{\beta}\Omega, t)} \sum_{\beta} P_{\Omega}(\tau^{\beta}\Omega, t) \ \sum_{\beta'} P_{\Omega}(\tau^{(\beta'-\beta)}\Omega', t + \Delta t|\Omega, t) \\
&= \frac{1}{\sum_{\beta} P_{\Omega}(\tau^{\beta}\Omega, t)} \sum_{\beta} P_{\Omega}(\tau^{\beta}\Omega, t) \ \sum_{\beta'} P_{\Omega}(\tau^{\beta'}\Omega', t + \Delta t|\Omega, t) \\
&= \sum_{\beta'} P_{\Omega}(\tau^{\beta'}\Omega', t + \Delta t|\Omega, t) \\
&= \sum_{\beta'} \nu_{\Omega}(\tau^{\beta'}\Omega', \Omega)\Delta t.
\end{aligned}$$

In the third equality, $\sum_{\beta} P_{\Omega}(\tau^{\beta}\Omega, t)$ is performed over all shifts β of an absolute configuration Ω such that its projection is the relative configuration ϑ i.e. $\Upsilon(\Omega) = \vartheta$. Similarly while determining $\displaystyle\sum_{\beta} \sum_{\beta'} P_{\Omega}(\tau^{\beta'}\Omega', t + \Delta t, \tau^{\beta}\Omega, t)$, the absolute configuration Ω' satisfies $\Upsilon(\Omega') = \vartheta'$.

In the sixth equality, translation invariance property is applied wherein both the absolute configurations at t and $t + \Delta t$ are shifted by β places to the left (via the operation $\tau^{-\beta}$). For the seventh equality, the set $\{\tau^{(\beta'-\beta)}\} = \{\tau^{\beta'}\}$, since β' is any integer and β is fixed. Moreover, since Ω has been arbitrarily chosen with the only condition that $\Upsilon(\Omega) = \vartheta$, this will hold for every $\Omega \in \Omega(\vartheta)$. Using the definition that $P_{\vartheta}(\vartheta', t + \Delta t|\vartheta, t) = \nu_{\vartheta}(\vartheta', \vartheta)\Delta t$,

$\nu_\vartheta(\vartheta', \vartheta)\Delta t = \sum_{\beta'} \nu_\Omega(\tau^{\beta'}\Omega', \Omega)\Delta t$ and thus,

$$\nu_\vartheta(\vartheta', \vartheta) = \sum_{\beta'} \nu_\Omega(\tau^{\beta'}\Omega', \Omega).$$

Since for a finite number of motors bound to a cargo the maximum distance between the vanguard and rearguard motor is finite (and equal to $\lceil \frac{n}{d} \rceil$ locations on the microtubule),

$$\nu_\vartheta(\vartheta', \vartheta) = \sum_{0 \leq \beta \leq \lceil \frac{n}{d} \rceil} \nu_\Omega(\tau^{\beta}\Omega', \Omega).$$
