# Supplementary material for: Interrogating Emergent Transport Properties for Molecular Motor Ensembles: A Semi-analytical Approach
Source: PLoS Comput Biol. 2016 Nov 3;12(11):e1005152. doi: 10.1371/journal.pcbi.1005152 (PMC5094777; doi:10.1371/journal.pcbi.1005152)
Supplement: S3 Text — (PDF) [file pcbi.1005152.s003.pdf]

Supporting Information Text S3

Average velocity of the cargo

Here, average velocity of the cargo is determined by undertaking the following steps :

Step I : The expected change in cargo position when the initial and final relative configurations at t and $t+\Delta t$ are restricted to being ϑ and ϑ' respectively, denoted by $d_{avg}(\vartheta', \vartheta)$, is obtained.

Step II : The expected change in the cargo position in time Δt, denoted as $D_{\Delta t}$ is obtained by removing the restriction of the relative configurations being ϑ and ϑ' at t and $t+\Delta t$. The average velocity of the cargo at time t follows as,

$$v(t) = \frac{D_{\Delta t}}{\Delta t}$$

To obtain $d_{avg}(\vartheta', \vartheta)$ the following steps are undertaken :

1. The change in cargo equilibrium position,

$$d(\Omega', \Omega) = Z_{eq}(\Omega') - Z_{eq}(\Omega),$$

   is determined for every possible transition from an initial absolute configuration Ω at time t to a final absolute configuration Ω' at time $t+\Delta t$, where Ω and Ω' are chosen such that $\Upsilon(\Omega) = \vartheta$ and $\Upsilon(\Omega') = \vartheta'$.

2. The probability $P(\Omega', t+\Delta t, \Omega, t|\vartheta', t+\Delta t, \vartheta, t)$ of transitioning from Ω to Ω' in time Δt is determined for every such pair of absolute configurations (Ω, Ω'), conditioned on the fact that the relative configuration also transitions from ϑ to ϑ' in the same time Δt.

3. A weighted sum of $d(\Omega', \Omega)$ with the weights given by the probabilities $P(\Omega' t+\Delta t, \Omega, t|\vartheta', t+\Delta t, \vartheta, t)$ is obtained.

Starting with any pair of absolute configurations Ω and Ω' satisfying the conditions $\Upsilon^{(e)}(\Omega) = \vartheta$ and $\Upsilon^{(e)}(\Omega') = \vartheta'$, the expected change in cargo position $d_{avg}(\vartheta', \vartheta)$, when the initial and final relative configurations at t and $t+\Delta t$ are restricted to being ϑ and ϑ' respectively, is given by

$$\begin{aligned}
d_{avg}(\vartheta', \vartheta) :&= \sum_{\Omega \in \Omega(\vartheta)} \sum_{\Omega' \in \Omega(\vartheta')} d(\Omega', \Omega) P(\Omega', t+\Delta t, \Omega, t|\vartheta', t+\Delta t, \vartheta, t) \\
&= \sum_{\beta} \sum_{\beta'} d(\tau^{\beta'}\Omega', \tau^{\beta}\Omega) P(\tau^{\beta'}\Omega', t+\Delta t, \tau^{\beta}\Omega, t|\vartheta', t+\Delta t, \vartheta, t) \\
&= \sum_{\beta} \sum_{\beta'} d(\tau^{\beta'}\Omega', \tau^{\beta}\Omega) \ \frac{P(\tau^{\beta'}\Omega', t+\Delta t, \tau^{\beta}\Omega, t, \vartheta', t+\Delta t, \vartheta, t)}{P(\vartheta', t+\Delta t, \vartheta, t)} \\
&= \sum_{\beta} \sum_{\beta'} d(\tau^{\beta'}\Omega', \tau^{\beta}\Omega) \ \frac{P(\tau^{\beta'}\Omega', t+\Delta t, \tau^{\beta}\Omega, t)}{P(\vartheta', t+\Delta t, \vartheta, t)} \\
&= \sum_{\beta} \sum_{\beta'} d(\tau^{\beta'}\Omega', \tau^{\beta}\Omega) \ \frac{\nu_{\Omega}(\tau^{\beta'}\Omega', \tau^{\beta}\Omega) P_{\Omega}(\tau^{\beta}\Omega, t)}{\nu_{\vartheta}(\vartheta', \vartheta) P_{\vartheta}(\vartheta, t)} \\
&= \frac{1}{\nu_{\vartheta}(\vartheta', \vartheta) P_{\vartheta}(\vartheta, t)} \sum_{\beta} P_{\Omega}(\tau^{\beta}\Omega, t) \ \sum_{\beta'} d(\tau^{\beta'}\Omega', \tau^{\beta}\Omega) \nu_{\Omega}(\tau^{\beta'}\Omega', \tau^{\beta}\Omega)
\end{aligned}$$

$$
\begin{aligned}
&= \frac{1}{\nu_\vartheta(\vartheta',\vartheta)P_\vartheta(\vartheta,t)}\sum_{\beta} P_\Omega(\tau^\beta\Omega,t)\ \sum_{\beta'} d(\tau^{(\beta'-\beta)}\Omega',\Omega)\nu_\Omega(\tau^{(\beta'-\beta)}\Omega',\Omega)\\
&= \frac{1}{\nu_\vartheta(\vartheta',\vartheta)P_\vartheta(\vartheta,t)}\sum_{\beta} P_\Omega(\tau^\beta\Omega,t)\ \sum_{\beta'} d(\tau^{\beta'}\Omega',\Omega)\nu_\Omega(\tau^{\beta'}\Omega',\Omega)\\
&= \frac{1}{\nu_\vartheta(\vartheta',\vartheta)}\sum_{\beta'} d(\tau^{\beta'}\Omega',\Omega)\nu_\Omega(\tau^{\beta'}\Omega',\Omega).
\end{aligned}
$$

In the seventh equality, translation invariance property is applied wherein both the absolute configurations at t and $t+\Delta t$ are shifted by β places to the left (via. the operation $\tau^{-\beta}$). For the eighth equality, the set $\{\tau^{(\beta'-\beta)}\} = \{\tau^{\beta'}\}$, since β' is any integer and β is fixed. The result is identical for any choice of absolute configuration Ω that satisfies $\Upsilon^{(e)}(\Omega) = \vartheta$. Thus,

$$
d_{avg}(\vartheta',\vartheta) = \frac{1}{\nu_\vartheta(\vartheta',\vartheta)}\sum_{\beta'} d(\tau^{\beta'}\Omega',\Omega)\nu_\Omega(\tau^{\beta'}\Omega',\Omega).
$$

After obtaining the expected value $d_{avg}(\vartheta',\vartheta)$, the expected change $D_{\Delta t}$ in cargo position in time Δt is determined to be,

$$
\begin{aligned}
D_{\Delta t} &= \sum_{\vartheta\in H}\sum_{\vartheta'\in H} d_{avg}(\vartheta',\vartheta)P_\vartheta(\vartheta',t+\Delta t,\vartheta,t),\\
&= \sum_{\vartheta\in H}\sum_{\vartheta'\in H} d_{avg}(\vartheta',\vartheta)\nu_\vartheta(\vartheta',\vartheta)P_\vartheta(\vartheta,t)(\Delta t).
\end{aligned}
$$

where H is the set of all possible relative configurations. It enables the calculation of average velocity as,

$$
\begin{aligned}
v(t) &= \frac{D_{\Delta t}}{\Delta t}\\
&= \sum_{\vartheta\in H}\sum_{\vartheta'\in H} d_{avg}(\vartheta',\vartheta)\nu_\vartheta(\vartheta',\vartheta)P_\vartheta(\vartheta,t).
\end{aligned}
$$
