# Supplementary material for: Interrogating Emergent Transport Properties for Molecular Motor Ensembles: A Semi-analytical Approach
Source: PLoS Comput Biol. 2016 Nov 3;12(11):e1005152. doi: 10.1371/journal.pcbi.1005152 (PMC5094777; doi:10.1371/journal.pcbi.1005152)
Supplement: S3 Fig — (PDF) [file pcbi.1005152.s006.pdf]

Supporting Information Figure S3

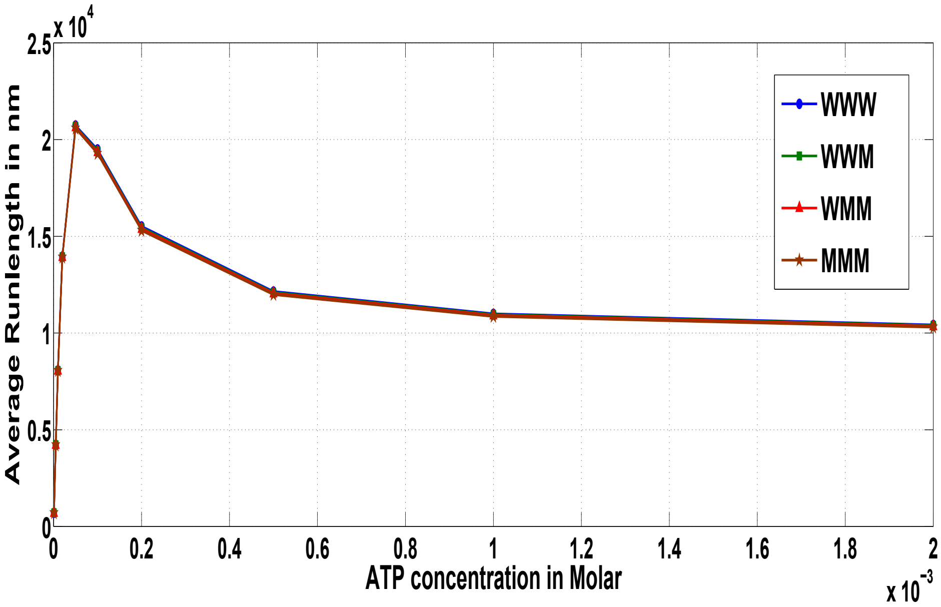

Figure 1: Effect of ATP concentration on average runlength for 3-motor ensembles WWW, WWM, WMM and MMM against load force of 0.2 pN
